# Therapeutic Response to Myosin Inhibitor Therapy in Noonan Syndrome–Associated Obstructive Hypertrophic Cardiomyopathy

**DOI:** 10.1016/j.jaccas.2025.106223

**Published:** 2025-12-04

**Authors:** Athanasios Feidakis, Richard Nies, Merve Kural, Lenhard Pennig, Nora Winnerling, Florian Erger, Roman Pfister, Katharina Seuthe

**Affiliations:** a Department III of Internal Medicine, Faculty of Medicine and University Hospital Cologne, University of Cologne, Cologne, Germany; b Institute for Diagnostic and Interventional Radiology, Faculty of Medicine and University Hospital Cologne, University of Cologne, Cologne, Germany; c Department of Human Genetics, Faculty of Medicine and University Hospital Cologne, University of Cologne, Cologne, Germany

**Keywords:** cardiomyopathy, genetic disorders, left ventricle, phenotype

## Abstract

**Background:**

Myosin inhibitor therapy is a novel option for hypertrophic cardiomyopathy (HCM) with left ventricular outflow tract obstruction. Noonan syndrome (NS), a RASopathy, can mimic HCM but was excluded from myosin inhibitor trials.

**Case Summary:**

A 60-year-old woman with obstructive HCM (HOCM) presented with progressive dyspnea (NYHA functional class III). Echocardiography showed severe septal hypertrophy (19 mm) and persistent left ventricular outflow tract obstruction (peak gradient: 51 mm Hg during Valsalva) despite beta-blocker therapy. Mavacamten, a myosin ATPase inhibitor, was initiated. After 6 months, symptoms improved (NYHA functional class II), the gradient decreased to 11 mm Hg, and N-terminal pro–B-type natriuretic peptide levels normalized. Genetic testing later confirmed NS.

**Discussion:**

This is to our knowledge the first reported case of myosin inhibitor efficacy in NS-associated HOCM. Although NS-related hypertrophy stems from RAS/MAPK pathway dysregulation, the patient responded similarly to those with sarcomeric mutations. This suggests downstream hypercontractility may be a shared therapeutic target.

**Take-Home Messages:**

Myosin inhibitors may benefit syndromic HOCM. Further research is warranted.

## Graphical Abstract

**Figure:**
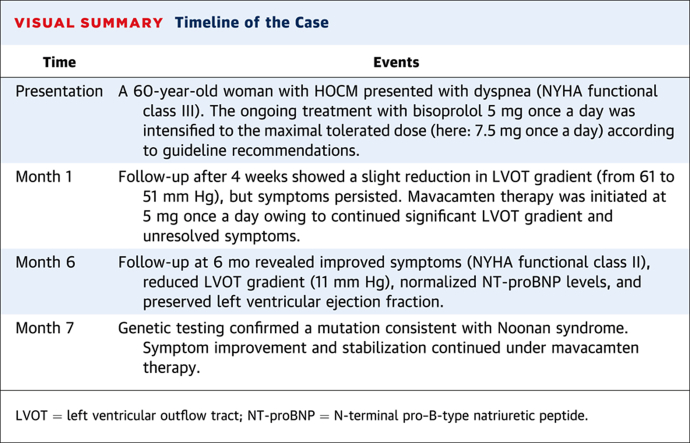

## History of Presentation

A 60-year-old woman with a history of symptomatic hypertrophic obstructive cardiomyopathy (HOCM) presented to our tertiary referral center for special consultation. The disease was initially diagnosed in 2023, and the patient had been treated since then with a beta-blocker (bisoprolol 5 mg once daily). However, she had experienced a slow but steady deterioration of her shortness of breath, which at the time of presentation was classified as NYHA functional class III. She did not report dizziness or angina pectoris.

Clinical examination revealed a blood pressure of 129/77 mm Hg, mild bradycardia (heart rate: 54 beats/min), and a normal body habitus without syndromic features such as hypertelorism, low-set posteriorly rotated ears, or a webbed neck. Apart from a grade 3/6 systolic ejection murmur at the left upper sternal border, the rest of the clinical examination was unremarkable.

## Past Medical History

Besides the known HOCM, the patient had bronchial asthma and arterial hypertension treated with amlodipine 5 mg once daily. Notable findings in the family history included the unexplained death of the patient's mother at the age of 40 and a congenital heart defect in the patient's older daughter, which was surgically corrected at the age of 7. The patient's father, 6 siblings, and younger daughter were reported to be in good health.

## Investigations

**Figure 1:**
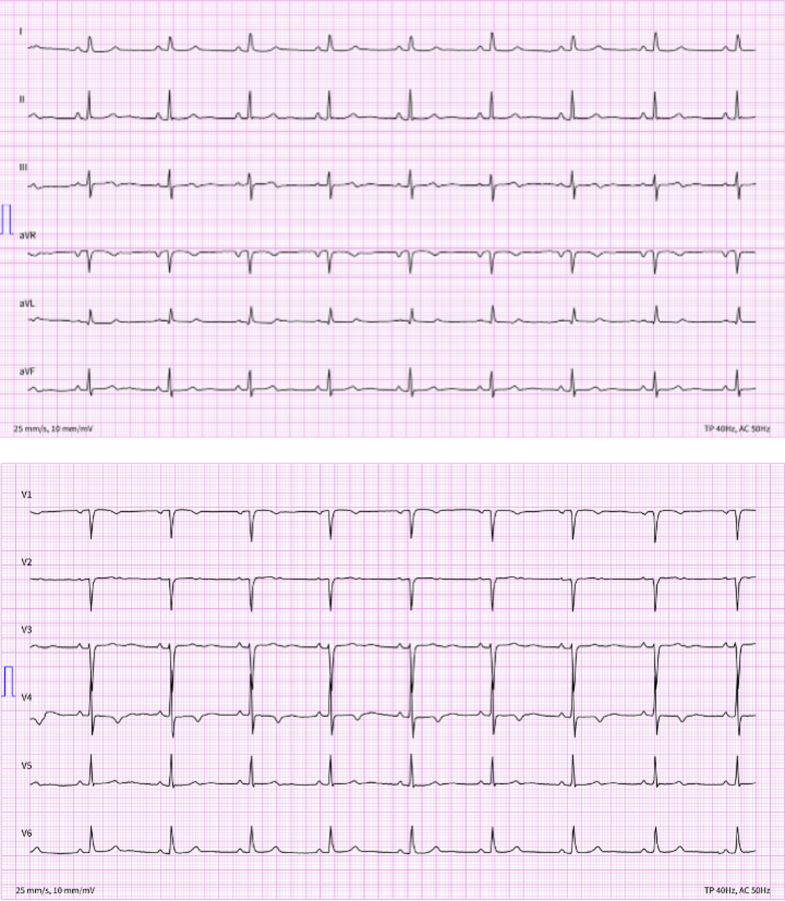
Resting 12-Lead Electrocardiogram at the Time of Admission

Electrocardiogram showed a bradycardic sinus rhythm (heart rate: 54 beats/min) with T-wave inversion in V1 and V4, a pseudo-Q wave in V1, and flattened/biphasic T waves in V2, but without any signs of left ventricular hypertrophy (LVH) (Figure 1).

Transthoracic echocardiography revealed severe concentric LVH with an interventricular septal thickness in end-diastole of 19 mm, a normal left ventricular ejection fraction (Video 1), systolic anterior motion of the mitral valve (Video 2) with subsequent moderate insufficiency (Video 3), and left ventricular outflow tract obstruction (LVOTO) with a peak gradient of 61 mm Hg during the Valsalva maneuver. Furthermore, diastolic indices indicated impaired relaxation (E/A: 0.62, lateral e′: 7 cm/s, septal e′: 4 cm/s) accompanied by mild left atrial enlargement (left atrial volume index: 34.8 mL/m^2^) but without evidence of abnormally elevated left ventricular filling pressure (E/e′: 8.97).

Blood tests showed normal renal function and an elevated N-terminal pro–B-type natriuretic peptide (NT-proBNP) value of 641 ng/L. There was no evidence of monoclonal gammopathy or Bence-Jones proteinuria. Genetic testing was performed after informed consent.

**Figure 2:**
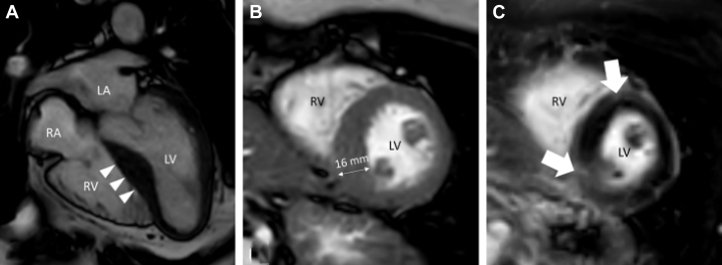
Findings on Cardiac Magnetic Resonance Cardiac magnetic resonance balanced steady-state free precession cine in (A) 4-chamber and (B) short-axis views and (C) late gadolinium enhancement in short-axis view. Images depict the manifestations of obstructive hypertrophic cardiomyopathy with asymmetric hypertrophy of the basal and midventricular interventricular septum (arrowheads in A) yielding a maximal wall thickness of 16 mm (B) and replacement fibrosis at the right ventricular insertion points (arrows in C). LA = left atrium; LV = left ventricle; RA = right atrium; RV = right ventricle.

Cardiac magnetic resonance demonstrated a septal-dominant LVH with replacement fibrosis at the insertion points of the right ventricle as depicted on late gadolinium enhancement (2% of left ventricular mass), accompanied by global interstitial fibrosis as delineated by prolonged T1 relaxation times (Figure 2, Video 4). These findings were inconsistent with an infiltrative disorder and suggestive of hypertrophic cardiomyopathy (HCM).

## Management

**Figure 3:**
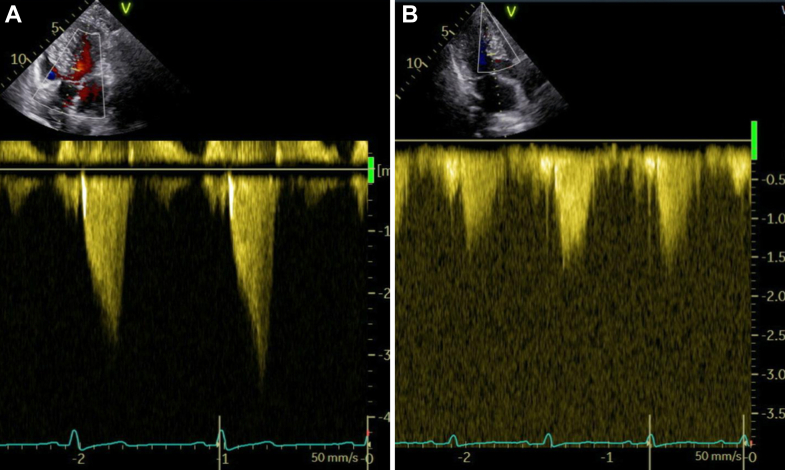
Baseline and Follow-Up LVOT Gradients on Transthoracic Echocardiography LVOT gradient during the Valsalva maneuver (A) at baseline and (B) at the 6-month follow-up. LVOT = left ventricular outflow tract.

According to guidelines recommendations,^1^ we first up-titrated the beta-blocker dose to the maximal tolerated level (in this case, 7.5 mg bisoprolol once daily given symptomatic bradycardia) and reassessed the patient after 4 weeks. Because of a persistently elevated left ventricular outflow tract (LVOT) gradient (51 mm Hg during the Valsalva maneuver, Figure 3A) and ongoing symptoms (NYHA functional class III), we decided to initiate additional treatment with the first-in-class myosin inhibitor mavacamten. As the patient was a normal CYP2C19 metabolizer, the starting dose was 5 mg once daily as per manufacturer recommendations. Intensification of therapy with disopyramide was not feasible owing to symptomatic bradycardia and limited availability in Germany.

## Outcome and Follow-Up

The patient was evaluated every 4 weeks at our outpatient clinic. A constant improvement in symptoms, laboratory values, and echocardiographic findings was observed throughout the myosin inhibitor therapy, with the peak LVOT gradient normalizing at 3 months after mavacamten initiation (14 mm Hg during the Valsalva maneuver).

At the 6-month follow-up, dyspnea was classified as NYHA functional class II, the peak LVOT gradient during the Valsalva maneuver was 11 mm Hg (Figure 3B), and NT-proBNP notably decreased to 202 ng/L. A comparison of NYHA class, peak LVOT gradient, and NT-proBNP values before mavacamten initiation and after 6 months of therapy is presented in Table 1. The systolic function of the left ventricle remained within the normal range (Video 5). Neither systolic anterior motion nor regurgitation of the mitral valve were detectable any longer (Video 6, Video 7). Additionally, a marked reduction in the left atrial volume index was observed (22.3 mL/m^2^), indicating favorable reverse remodeling. A detailed comparison of the remaining diastolic function parameters at baseline and after mavacamten treatment is shown in Table 2. Given the reduction in the LVOT gradient, no further escalation of the mavacamten dose was warranted, which was sustained at 5 mg once daily throughout the course of therapy. The patient remained normotensive, and no adjustment of antihypertensive therapy was required.

**Table 1:** Clinical, Laboratory and Echocardiographic Values Before and 6 Months After Treatment With Mavacamten

	Pretreatment	6-mo Follow-Up
NYHA functional class	III	II
Peak LVOT gradient during the Valsalva maneuver (mm Hg)	51	11
NT-proBNP (ng/L)	641	202
LVEF (%)	65	59

LVEF = left ventricular ejection fraction; LVOT = left ventricular outflow tract; NT-proBNP = N-terminal pro–B-type natriuretic peptide.

**Table 2:** Direct Comparison of Echocardiographic Diastolic Function Parameters Before and 6 Months After Treatment With Mavacamten

	Pretreatment	6-mo Follow-Up
LAVI (mL/m^2^)	34.8	22.3
E/A	0.62	0.74
e′ lateral (cm/s)	7	8
e′ medial (cm/s)	4	5
E/e′	8.97	9.82
TRV_max_ (m/s)	2.27	2.4

LAVI = left atrial volume index; TRV_max_ = maximum tricuspid regurgitation velocity.

Seven months after initiating therapy with a myosin inhibitor, gene panel next-generation sequencing revealed a heterozygous pathogenic variant in the *PTPN11* gene (c.1403C>T; p.Thr468Met) on chromosome 12, consistent with a molecular genetic diagnosis of Noonan syndrome (NS). No pathogenic or likely pathogenic variants were found in 216 other genes associated with pediatric-onset or adult-onset monogenic cardiomyopathies, including syndromic forms.

## Discussion

HCM is an inherited heart disease, characterized predominantly by LVH in the absence of another cardiac, systemic, or metabolic disease capable of producing the magnitude of hypertrophy evident in a given patient and for which a disease-causing sarcomere (or sarcomere-related) variant is identified or genetic etiology remains unresolved.^1^

NS is an autosomal dominant genetic disorder grouped among the RASopathies, a family of developmental multisystemic disorders caused by germline variants in the RAS (rat sarcoma)/MAPK (mitogen-activated protein kinase) pathway.^2^ Cardiovascular involvement occurs in 80 to 90% of NS cases, most often presenting as congenital heart defect–like pulmonic valve stenosis and/or early-onset HCM, with the latter affecting approximately 20% of individuals with NS.^3^

The molecular mechanisms driving hypertrophy in RASopathies involve complex signaling pathways and depend on the underlying gene variant.^2^ Variants causing NS typically lead to upregulation of the RAS/MAPK pathway, while specific variants, such as those in *RAF1* or *PTPN11*, drive enhanced PI3K-AKT-mTOR signaling.^4^ However the myocardial histology in the RASopathies-associated HCM is indistinguishable from that observed in sarcomeric HCM.^5^

In sarcomeric HCM, on the other hand, cardiac contractility is increased because of mutations that disrupt the sarcomere equilibrium: More myosin heads are accessible to form cross-bridges with actin, while fewer myosin molecules remain in their energy-saving super-relaxed state.^6^ Current guideline-recommended treatments^1^ primarily target downstream mechanisms to reduce hypercontractility, but these approaches rely heavily on observational studies^7^^,^^8^ and often fall short in fully alleviating symptoms.

Novel therapies such as cardiac myosin ATPase inhibitors target the primary pathophysiological abnormality associated with sarcomeric mutations—that is, hypercontractility^9^—and offer new therapeutic options for symptomatic patients with LVOTO. However, patients with known syndromic disorders that mimic HOCM (such as NS with LVH) were excluded from the major phase III trials of mavacamten.^10^^,^^11^

To our knowledge, this represents the first documented case of a patient with NS-associated HOCM receiving myosin inhibitor treatment. The remarkable clinical improvement observed in alleviating LVOTO highlights the potential of myosin inhibitor therapy, extending its applicability beyond sarcomeric HCM to nonsarcomeric forms associated with genetic syndromes such as RASopathies.

Supporting this notion, a recent preprint discusses a potential therapeutic effect of mavacamten in RASopathy-associated HCM.^12^ In vitro data from iPSC- derived cardiomyocytes harboring BRAF-RASopathy mutations demonstrated improved mitochondrial function under mavacamten treatment.^12^

## Conclusions

Despite the distinct molecular mechanisms underlying hypertrophy in sarcomeric HCM and NS-associated HCM, the reduction in contractility and diastolic stiffening induced by myosin ATPase inhibition appears to be effective in both clinical entities. However, further studies are needed to investigate the long-term efficacy and safety of this treatment approach, as well as to better understand its potential impact across diverse patient populations.

## Funding Support and Author Disclosures

Drs Pfister and Seuthe have been on the Speakers Bureau for Bristol Myers Squibb. All other authors have reported that they have no relationships relevant to the contents of this paper to disclose.

**Take-Home Messages**

• Myosin inhibitors such as mavacamten may effectively reduce LVOTO and alleviate symptoms in syndromic HCM such as that seen in NS, despite differing underlying molecular mechanisms.
• The successful use of mavacamten in this case highlights the potential to expand its therapeutic applications beyond sarcomeric HCM to other nonsarcomeric forms of HCM associated with genetic syndromes.

## Appendix

Video 1 Apical 4-Chamber View Before Myosin Inhibitor Treatment

Video 2 Zoomed Apical 3-Chamber View Before Myosin Inhibitor Treatment Demonstrating SAM of the Mitral Valve

Video 3 Apical 2-Chamber View With Color Doppler Before Myosin Inhibitor Treatment Showing Mitral Regurgitation

Video 4 Cardiac Magnetic Resonance, 4-Chamber View Before Myosin Inhibitor Treatment

Video 5 Apical 4-Chamber View After Myosin Inhibitor Treatment

Video 6 Zoomed Apical 4-Chamber View After Myosin Inhibitor Treatment Demonstrating Absence of SAM of the Mitral Valve

Video 7 Apical 4-Chamber View With Color Doppler After Myosin Inhibitor Treatment Demonstrating Absence of Mitral Regurgitation

## References

[1] Ommen S., Ho C., Asif I. (2024). 2024 AHA/ACC/AMSSM/HRS/PACES/SCMR Guideline for the management of hypertrophic cardiomyopathy: a report of the American Heart Association/American College of Cardiology joint committee on clinical practice guidelines. J Am Coll Cardiol.

[2] Lioncino M., Monda E., Verrillo F. (2022). Hypertrophic cardiomyopathy in RASopathies: diagnosis, clinical characteristics, prognostic implications, and management. Heart Fail Clin.

[3] Maron B.J., Towbin J.A., Thiene G. (2006). Contemporary definitions and classification of the cardiomyopathies: an American Heart Association Scientific Statement from the Council on Clinical Cardiology, Heart Failure and Transplantation Committee; Quality of Care and Outcomes Research and Functional Genomics and Translational Biology Interdisciplinary Working Groups; and Council on Epidemiology and Prevention. Circulation.

[4] Tartaglia M., Gelb B.D. (2010). Disorders of dysregulated signal traffic through the RAS-MAPK pathway: phenotypic spectrum and molecular mechanisms. Ann N Y Acad Sci.

[5] Burch M., Mann J.M., Sharland M., Shinebourne E.A., Patton M.A., McKenna W.J. (1992). Myocardial disarray in Noonan syndrome. Br Heart J.

[6] Toepfer C.N., Wakimoto H., Garfinkel A.C. (2019). Hypertrophic cardiomyopathy mutations in MYBPC3 dysregulate myosin. Sci Transl Med.

[7] Iavarone M., Monda E., Vritz O. (2022). Medical treatment of patients with hypertrophic cardiomyopathy: an overview of current and emerging therapy. Arch Cardiovasc Dis.

[8] Monda E., Lioncino M., Palmiero G. (2022). Bisoprolol for treatment of symptomatic patients with obstructive hypertrophic cardiomyopathy. The BASIC (bisoprolol AS therapy in hypertrophic cardiomyopathy) study. Int J Cardiol.

[9] Zampieri M., Berteotti M., Ferrantini C. (2021). Pathophysiology and treatment of hypertrophic cardiomyopathy: new perspectives. Curr Heart Fail Rep.

[10] Olivotto I., Oreziak A., Barriales-Villa R., EXPLORER-HCM study investigators (2020). Mavacamten for treatment of symptomatic obstructive hypertrophic cardiomyopathy (EXPLORER-HCM): a randomised, double-blind, placebo-controlled, phase 3 trial. Lancet.

[11] Desai M.Y., Owens A., Geske J.B. (2022). Myosin inhibition in patients with obstructive hypertrophic cardiomyopathy referred for septal reduction therapy. J Am Coll Cardiol.

[12] Ruiz-Velasco A., Jouve C., Deshayes L., Kohlhaas M., Maack C., Hulot J.S. (2025). Mavacamten improves energy balance in a pre-clinical model of RASopathy-associated hypertrophic cardiomyopathy. bioRxiv.

